# Bounds for the M-spectral radius of a fourth-order partially symmetric tensor

**DOI:** 10.1186/s13660-018-1610-5

**Published:** 2018-01-12

**Authors:** Suhua Li, Yaotang Li

**Affiliations:** School of Mathematics and Statistics, Yunnan University, Kunming, Yunnan 650091 P.R. China

**Keywords:** M-eigenvalues, M-spectral radius, partially symmetric, tensors

## Abstract

M-eigenvalues of fourth-order partially symmetric tensors play an important role in many real fields such as quantum entanglement and nonlinear elastic materials analysis. In this paper, we give two bounds for the maximal absolute value of all the M-eigenvalues (called the M-spectral radius) of a fourth-order partially symmetric tensor and discuss the relation of them. A numerical example is given to explain the proposed results.

## Introduction

A fourth-order real tensor $\mathcal{A}=(a_{i_{1}i_{2}i_{3}i_{4}})\in \mathbb{R}^{m\times n\times m\times n}$ is called partially symmetric [1] if it has the following symmetry:

$$a_{i_{1}i_{2}i_{3}i_{4}}=a_{i_{3}i_{2}i_{1}i_{4}}=a_{i_{1}i_{4}i_{3}i_{2}}, \quad\forall i_{1}, i_{3}\in[m], \forall i_{2}, i_{4}\in[n], $$

where $[m]=\{1,2,\ldots,m\}$ and $[n]=\{1,2,\ldots,n\}$. Such a tensor often arises in nonlinear elastic materials analysis [2, 3] and entanglement studies in quantum physics [4–6]. For this tensor, there are many kinds of eigenvalues such as H-eigenvalues, Z-eigenvalues, and D-eigenvalues [7, 8]; here we only discuss its M-eigenvalues [1, 9].

### Definition 1

([9])

Let $\mathcal{A}=(a_{i_{1}i_{2}i_{3}i_{4}})\in\mathbb{R}^{m\times n\times m\times n}$ be a partially symmetric tensor, and let $\lambda \in\mathbb{R}$. Suppose that there are real vectors $x\in\mathbb {R}^{m}$ and $y\in\mathbb{R}^{n}$ such that

1 $$ \left \{ \textstyle\begin{array}{l} \mathcal{A}\cdot yxy=\lambda x,\\ \mathcal{A}xyx\cdot=\lambda y,\\ x^{T}x=1,\\ y^{T}y=1, \end{array}\displaystyle \right . $$

where $\mathcal{A}\cdot yxy\in\mathbb{R}^{m}$ and $\mathcal{A}xyx\cdot \in\mathbb{R}^{n}$ with *i*th components

$$ (\mathcal{A}\cdot yxy)_{i}=\sum_{i_{3}\in[m]}\sum _{i_{2},i_{4}\in [n]}a_{ii_{2}i_{3}i_{4}}y_{i_{2}}x_{i_{3}}y_{i_{4}} $$

and

$$ (\mathcal{A}xyx\cdot)_{i}=\sum_{i_{1},i_{3}\in[m]}\sum _{i_{2}\in [n]}a_{i_{1}i_{2}i_{3}i}x_{i_{1}}y_{i_{2}}x_{i_{3}}. $$

Then *λ* is called an M-eigenvalue of $\mathcal{A}$ with left M-eigenvector *x* and right M-eigenvector *y*.

Note that M-eigenvalues of a fourth-order partially symmetric tensor always exist [1]. They have a close relation to many problems in the theory of elasticity and quantum physics [1, 9, 10]. For example, the largest M-eigenvalue of $\mathcal {A}=(a_{i_{1}i_{2}i_{3}i_{4}})\in\mathbb{R}^{m\times n\times m\times n}$, denoted by

$$ \lambda^{\star}=\max\{\lambda: \lambda \text{ is an M-eigenvalue of } \mathcal{A}\}, $$

is the optimum solution of the problem (for details, see [9])

$$ \begin{gathered} \text{max} \quad f(x, y)=\sum _{i_{1},i_{3}=1}^{m}\sum_{i_{2},i_{4}=1}^{n}a_{i_{1}i_{2}i_{3}i_{4}}x_{i_{1}}y_{i_{2}}x_{i_{3}}y_{i_{4}}, \\ \quad\text{s.t.} \quad x^{T}x=1, \qquad y^{T}y=1, \quad x\in \mathbb {R}^{m}, y\in\mathbb{R}^{n}. \end{gathered} $$

The outer product $\lambda x\circ y\circ x\circ y$, where

$$ (\lambda x\circ y\circ x\circ y)_{i_{1}i_{2}i_{3}i_{4}}=\lambda x_{i_{1}}y_{i_{2}}x_{i_{3}}y_{i_{4}},\quad \forall i_{1}, i_{3}\in[m], \forall i_{2}, i_{4}\in[n] $$

and *λ* is an M-eigenvalue with the maximal absolute value of $\mathcal{A}=(a_{i_{1}i_{2}i_{3}i_{4}})\in\mathbb{R}^{m\times n\times m\times n}$ with left M-eigenvector $x\in\mathbb{R}^{m}$ and right M-eigenvector $y\in\mathbb{R}^{n}$, is a partially symmetric best rank-one approximation of $\mathcal {A}$ [1], which has wide applications in signal and image processing, wireless communication systems, and independent component analysis [11–14]. The M-spectral radius of $\mathcal{A}=(a_{i_{1}i_{2}i_{3}i_{4}})\in \mathbb{R}^{m\times n\times m\times n}$, denoted by

$$ \rho_{{\mathrm{M}}}(\mathcal{A})=\max\bigl\{ |\lambda|: \lambda \text{ is an M-eigenvalue of } \mathcal{A}\bigr\} , $$

has significant impacts on identifying nonsingular $\mathscr {M}$-tensors, which satisfy the strong ellipticity condition [10].

To our knowledge, there are few results about bounds for the M-spectral radius of a fourth-order partially symmetric tensor. In this paper, we present two bounds for the M-spectral radius and discuss their relation. A numerical example is also given to explain the proposed results.

## Two bounds for the M-spectral radius

In this section, we give two bounds for the M-spectral radius of fourth-order partially symmetric tensors and discuss their relation.

### Theorem 1

*Let* $\mathcal{A}=(a_{i_{1}i_{2}i_{3}i_{4}})\in\mathbb{R}^{m\times n\times m\times n}$ *be a partially symmetric tensor*. *Then*

2 $$ \rho_{{\mathrm{M}}}(\mathcal{A})\leq\sqrt{\max _{i\in[m]}\bigl\{ R_{i}(\mathcal{A})\bigr\} \cdot\max _{l\in[n]}\bigl\{ C_{l}(\mathcal{A})\bigr\} }, $$

*where*

$$ R_{i}(\mathcal{A}) =\sum_{i_{3}\in[m]}\sum _{i_{2}, i_{4}\in [n]}|a_{ii_{2}i_{3}i_{4}}|, \qquad C_{l}( \mathcal{A})=\sum_{i_{1}, i_{3}\in [m]}\sum _{i_{2}\in[n]}|a_{i_{1}i_{2}i_{3}l}|. $$

### Proof

Suppose that *λ* is an M-eigenvalue of $\mathcal{A}$ and that $x\in\mathbb{R}^{m}$ and $y\in\mathbb{R}^{n}$ are associated left M-eigenvector and right M-eigenvector. Then (1) holds. Let

$$ x_{p}=\max_{k\in[m]}\bigl\{ |x_{k}|\bigr\} ,\qquad y_{q}=\max_{k\in[n]}\bigl\{ |y_{k}|\bigr\} . $$

Since $x^{T}x=1$ and $y^{T}y=1$, we have

3 $$ 0< |x_{p}|\leq1,\qquad 0< |y_{q}|\leq1. $$

The *p*th equation of $\mathcal{A}\cdot yxy=\lambda x$ is

4 $$ \lambda x_{p}=\sum_{i_{3}\in[m]}\sum _{i_{2},i_{4}\in [n]}a_{pi_{2}i_{3}i_{4}}y_{i_{2}}x_{i_{3}}y_{i_{4}}. $$

Taking the absolute values on both sides of (4) and using the triangle inequality give

5 $$ \begin{aligned}[b] |\lambda||x_{p}|&\leq\sum _{i_{3}\in[m]}\sum_{i_{2},i_{4}\in [n]}|a_{pi_{2}i_{3}i_{4}}||y_{i_{2}}||x_{i_{3}}||y_{i_{4}}| \\ &\leq\sum_{i_{3}\in[m]}\sum_{i_{2},i_{4}\in [n]}|a_{pi_{2}i_{3}i_{4}}||y_{q}| \\ &=R_{p}(\mathcal{A})|y_{q}|. \end{aligned} $$

Similarly, by the *q*th equation of $\mathcal{A}xyx\cdot=\lambda y$ we have

6 $$ \begin{aligned}[b] |\lambda||y_{q}|&\leq\sum _{i_{1},i_{3}\in[m]}\sum_{i_{2}\in [n]}|a_{i_{1}i_{2}i_{3}q}||x_{i_{1}}||y_{i_{2}}||x_{i_{3}}| \\ &\leq\sum_{i_{1},i_{3}\in[m]}\sum_{i_{2}\in [n]}|a_{i_{1}i_{2}i_{3}q}||x_{p}| \\ &=C_{q}(\mathcal{A})|x_{p}|. \end{aligned} $$

Multiplying (5) and (6) gives

$$ |\lambda|^{2}|x_{p}||y_{q}|\leq R_{p}(\mathcal{A})C_{q}(\mathcal {A})|x_{p}||y_{q}|, $$

which, together with (3), yields

7 $$ |\lambda|^{2}\leq R_{p}(\mathcal{A})C_{q}( \mathcal{A})\leq\max_{i\in [m],l\in[n]}\bigl\{ R_{i}( \mathcal{A})C_{l}(\mathcal{A})\bigr\} . $$

Since (7) holds for all M-eigenvalues of $\mathcal{A}$, we have

$$\rho_{{\mathrm{M}}}(\mathcal{A})\leq\sqrt{\max_{ i\in[m], l\in[n]}\bigl\{ R_{i}(\mathcal{A})C_{l}(\mathcal{A})\bigr\} }=\sqrt{\max _{i\in[m]}\bigl\{ R_{i}(\mathcal{A})\bigr\} \cdot\max _{l\in[n]}\bigl\{ C_{l}(\mathcal{A})\bigr\} }, $$

and the conclusion follows. □

### Theorem 2

*Let* $\mathcal{A}=(a_{i_{1}i_{2}i_{3}i_{4}})\in\mathbb{R}^{m\times n\times m\times n}$ *be a partially symmetric tensor*, *and let* *α* *be any subset of* $[m]$ *and* *β* *be any subset of* $[n]$. *Then*

8 $$ \rho_{{\mathrm{M}}}(\mathcal{A})\leq\min\{\mu_{1}, \mu_{2}\}, $$

*where*

$$\begin{gathered} \mu_{1}=\min_{\alpha\subseteq[m]} \biggl\{ \max _{p\in[m], q\in[n]} \biggl\{ \frac{1}{2} \bigl(R_{p}^{\alpha}( \mathcal{A})+\sqrt{R_{p}^{\alpha }(\mathcal{A})^{2}+4 \bigl(R_{p}(\mathcal{A})-R_{p}^{\alpha}(\mathcal {A}) \bigr)C_{q}(\mathcal{A})} \bigr) \biggr\} \biggr\} , \\ \mu_{2}=\min_{\beta\subseteq[n]} \biggl\{ \max_{p\in[m], q\in[n]} \biggl\{ \frac{1}{2} \bigl(C_{q}^{\beta}(\mathcal{A})+ \sqrt{C_{q}^{\beta }(\mathcal{A})^{2}+4 \bigl(C_{q}(\mathcal{A})-C_{q}^{\beta}(\mathcal {A}) \bigr)R_{p}(\mathcal{A})} \bigr) \biggr\} \biggr\} ,\end{gathered} $$

*and*

$$ R_{p}^{\alpha}(\mathcal{A})=\sum_{i_{3}\in\alpha} \sum_{i_{2},i_{4}\in [n]}|a_{pi_{2}i_{3}i_{4}}|,\qquad C_{q}^{\beta}( \mathcal{A})=\sum_{i_{2}\in \beta}\sum _{i_{1},i_{3}\in[m]}|a_{i_{1}i_{2}i_{3}q}|. $$

### Proof

Assume that *λ* is an M-eigenvalue of $\mathcal{A}$ and that $x\in\mathbb{R}^{m}$ and $y\in\mathbb{R}^{n}$ are the corresponding left M-eigenvector and right M-eigenvector. Then (1) holds. Let

$$ |x_{p}|=\max_{k\in[m]}\bigl\{ |x_{k}|\bigr\} ,\qquad |y_{q}|=\max_{k\in[n]}\bigl\{ |y_{k}|\bigr\} . $$

Then (3) holds. The *p*th equation of $\mathcal{A}\cdot yxy=\lambda x$ can be rewritten as

9 $$ \lambda x_{p}=\sum_{i_{3}\in\alpha} \sum_{i_{2}, i_{4}\in [n]}a_{pi_{2}i_{3}i_{4}}y_{i_{2}}x_{i_{3}}y_{i_{4}}+ \sum_{i_{3}\notin \alpha}\sum_{i_{2},i_{4}\in[n]}a_{pi_{2}i_{3}i_{4}}y_{i_{2}}x_{i_{3}}y_{i_{4}}. $$

By the technique for the inequality in Theorem 1, we obtain from (9) that

$$ \begin{aligned} |\lambda||x_{p}|&\leq\sum _{i_{3}\in\alpha}\sum_{i_{2}, i_{4}\in [n]}|a_{pi_{2}i_{3}i_{4}}||y_{i_{2}}||x_{p}||y_{i_{4}}|+ \sum_{i_{3}\notin\alpha}\sum_{i_{2},i_{4}\in [n]}|a_{pi_{2}i_{3}i_{4}}||y_{i_{2}}||x_{i_{3}}||y_{q}| \\ &\leq\sum_{i_{3}\in\alpha}\sum_{i_{2}, i_{4}\in [n]}|a_{pi_{2}i_{3}i_{4}}||x_{p}|+ \sum_{i_{3}\notin\alpha}\sum_{i_{2},i_{4}\in[n]}|a_{pi_{2}i_{3}i_{4}}||y_{q}| \\ &=R_{p}^{\alpha}(\mathcal{A})|x_{p}|+ \bigl(R_{p}(\mathcal{A})-R_{p}^{\alpha }(\mathcal{A}) \bigr)|y_{q}|, \end{aligned} $$

that is,

10 $$ \bigl(|\lambda|-R_{p}^{\alpha}(\mathcal{A}) \bigr)|x_{p}|\leq\bigl(R_{p}(\mathcal {A})-R_{p}^{\alpha}( \mathcal{A})\bigr)|y_{q}|. $$

In addition, by the *q*th equation of $\mathcal{A}xyx\cdot=\lambda y$ we have

11 $$ |\lambda||y_{q}|\leq\sum_{i_{1},i_{3}\in[m]} \sum_{i_{2}\in [n]}|a_{i_{1}i_{2}i_{3}q}||x_{p}|=C_{q}( \mathcal{A})|x_{p}|. $$

Multiplying (10) with (11) and using (3) yield

12 $$ \bigl(|\lambda|-R_{p}^{\alpha}(\mathcal{A}) \bigr)|\lambda|\leq\bigl(R_{p}(\mathcal {A})-R_{p}^{\alpha}( \mathcal{A})\bigr)C_{q}(\mathcal{A}). $$

Then

13 $$ \begin{aligned}[b] |\lambda|&\leq\frac{1}{2} \bigl(R_{p}^{\alpha}(\mathcal{A})+\sqrt{R_{p}^{\alpha}( \mathcal{A})^{2}+4\bigl(R_{p}(\mathcal{A}) -R_{p}^{\alpha}(\mathcal{A})\bigr)C_{q}(\mathcal{A})} \bigr) \\ &\leq\max_{p\in[m],q\in[n]} \biggl\{ \frac{1}{2} \bigl(R_{p}^{\alpha }(\mathcal{A})+\sqrt{R_{p}^{\alpha}( \mathcal{A})^{2}+ 4\bigl(R_{p}(\mathcal{A})-R_{p}^{\alpha}( \mathcal{A})\bigr)C_{q}(\mathcal {A})} \bigr) \biggr\} . \end{aligned} $$

Note that (13) holds for all M-eigenvalues of $\mathcal{A}$ and any $\alpha\subseteq[m]$. Hence

14 $$ \rho_{{\mathrm{M}}}(\mathcal{A})\leq\mu_{1}. $$

On the other hand, for the *q*th equation of $\mathcal{A}xyx\cdot =\lambda y$, we have

15 $$ \lambda y_{q}=\sum_{i_{2}\in\beta} \sum_{i_{1},i_{3}\in [m]}a_{i_{1}i_{2}i_{3}q}x_{i_{1}}y_{i_{2}}x_{i_{3}}+ \sum_{i_{2}\notin\beta}\sum_{i_{1},i_{3}\in [m]}a_{i_{1}i_{2}i_{3}q}x_{i_{1}}y_{i_{2}}x_{i_{3}}. $$

Then

$$ \begin{aligned} |\lambda||y_{q}|&\leq\sum _{i_{2}\in\beta}\sum_{i_{1},i_{3}\in [m]}|a_{i_{1}i_{2}i_{3}q}||y_{q}|+ \sum_{i_{2}\notin\beta}\sum_{i_{1},i_{3}\in[m]}|a_{i_{1}i_{2}i_{3}q}| |x_{p}| \\ &=C_{q}^{\beta}(\mathcal{A})|y_{q}|+ \bigl(C_{q}(\mathcal{A})-C_{q}^{\beta }(\mathcal{A}) \bigr)|x_{p}|, \end{aligned} $$

that is,

16 $$ \bigl(|\lambda|-C_{q}^{\beta}(\mathcal{A}) \bigr)|y_{q}|\leq\bigl(C_{q}(\mathcal {A})-C_{q}^{\beta}( \mathcal{A})\bigr)|x_{p}|. $$

By the *p*th equation of $\mathcal{A}\cdot yxy=\lambda x$ we have

17 $$ |\lambda||x_{p}|\leq\sum_{i_{3}\in[m]} \sum_{i_{2},i_{4}\in [n]}|a_{pi_{2}i_{3}i_{4}}||y_{q}|=R_{p}( \mathcal{A})|y_{q}|. $$

Multiplying (16) with (17) and using (3), we derive

18 $$ \bigl(|\lambda|-C_{q}^{\beta}(\mathcal{A}) \bigr)|\lambda|\leq\bigl(C_{q}(\mathcal {A})-C_{q}^{\beta}( \mathcal{A})\bigr)R_{p}(\mathcal{A}). $$

Hence

19 $$ \begin{aligned}[b] |\lambda|&\leq\frac{1}{2} \bigl(C_{q}^{\beta}(\mathcal{A})+\sqrt{C_{q}^{\beta}( \mathcal{A})^{2}+4\bigl(C_{q}(\mathcal{A})-C_{q}^{\beta }( \mathcal{A})\bigr)R_{p}(\mathcal{A})} \bigr) \\ &\leq\max_{p\in[m], q\in[n]} \biggl\{ \frac{1}{2} \bigl(C_{q}^{\beta }(\mathcal{A})+\sqrt{C_{q}^{\beta}( \mathcal{A})^{2}+4\bigl(C_{q}(\mathcal {A})-C_{q}^{\beta}( \mathcal{A})\bigr)R_{p}(\mathcal{A})} \bigr) \biggr\} . \end{aligned} $$

Since (19) holds for all M-eigenvalues of $\mathcal{A}$ and any $\beta\subseteq[n]$, we have

20 $$ \rho_{{\mathrm{M}}}(\mathcal{A})\leq\mu_{2}. $$

From (14) and (20) we have

$$ \rho_{{\mathrm{M}}}(\mathcal{A})\leq\min\{\mu_{1}, \mu_{2} \}. $$

The proof is completed. □

### Remark 1

Since

$$ \begin{gathered} \max_{p\in[m], q\in[n]} \biggl\{ \frac{1}{2} \bigl(R_{p}^{\alpha}(\mathcal {A})+ \sqrt{R_{p}^{\alpha}(\mathcal{A})^{2}+4 \bigl(R_{p}(\mathcal {A})-R_{p}^{\alpha}(\mathcal{A}) \bigr)C_{q}(\mathcal{A})} \bigr) \biggr\} \\ \quad=\max_{p\in[m], q\in[n]} \biggl\{ \frac{1}{2} \bigl(C_{q}^{\beta }(\mathcal{A})+\sqrt{C_{q}^{\beta}( \mathcal{A})^{2}+4\bigl(C_{q}(\mathcal {A})-C_{q}^{\beta}( \mathcal{A})\bigr)R_{p}(\mathcal{A})} \bigr) \biggr\} \\ \quad=\sqrt{\max_{p\in[m]}\bigl\{ R_{p}(\mathcal{A}) \bigr\} \cdot\max_{q\in [n]}\bigl\{ C_{q}(\mathcal{A})\bigr\} } \end{gathered} $$

when $\alpha=\varnothing$ and $\beta=\varnothing$, we have

$$ \min\{\mu_{1}, \mu_{2}\}\leq\sqrt{\max _{p\in[m]}\bigl\{ R_{p}(\mathcal {A})\bigr\} \cdot\max _{q\in[n]}\bigl\{ C_{q}(\mathcal{A})\bigr\} }. $$

Therefore, the bound in (8) is tighter than the bound in (2) for the M-spectral radius $\rho_{{\mathrm{M}}}(\mathcal{A})$ of a given tensor $\mathcal{A}$.

### Remark 2

Although the bound in (8) is tighter than the bound in (2), it is easier to compute the bound in (2) for the M-spectral radius of a given tensor.

Next, we use a numerical example to show the effectiveness of the bounds in Theorems 1 and 2.

### Example 1

Consider the partially symmetric tensor $\mathcal {A}_{1}=(a_{i_{1}i_{2}i_{3}i_{4}})\in\mathbb{R}^{3\times3\times 3\times3}$ with

$$ \begin{gathered} a_{1111}=1.1112,\qquad a_{1311}=6.1096,\qquad a_{3111}=0.3032,\qquad a_{2121}=1.4125, \\ a_{3131}=1,\qquad a_{1212}=0.0788,\qquad a_{2222}=1,\qquad a_{3222}=0.6032, \\ a_{3232}=0.3657,\qquad a_{1313}=2,\qquad a_{2323}=0.6226,\qquad a_{3333}=0.3, \end{gathered} $$

and the remaining zero elements. By Theorem 1 we have

$$ \rho_{{\mathrm{M}}}(\mathcal{A}_{1})\leq12.6843. $$

By Theorem 2 we have

$$ \rho_{{\mathrm{M}}}(\mathcal{A}_{1})\leq10.2397. $$

In fact, $\rho_{{\mathrm{M}}}(\mathcal{A}_{1})\approx7.6841$.

## Conclusions

In this paper, we have presented two bounds for the M-spectral radius of a fourth-order partially symmetric tensor and have indicated their relation. To show the effectiveness of the proposed results, a numerical example is also given.
